# A Retrospective Study of Ectoparasitosis in Patients Referred to Imam Reza Hospital of Mashhad, Iran

**DOI:** 10.1155/2014/104018

**Published:** 2014-04-22

**Authors:** Fariba Berenji, Narges Marvi-Moghadam, Parisa Naghibozakerin Meibodi

**Affiliations:** Department of Parasitology and Mycology, Medical Faculty of Mashhad University of Medical Sciences, Mashhad 917794-8564, Iran

## Abstract

This cross-sectional study was performed on all patients suspected to be suffering from ectoparasites who were referred to the parasitology laboratory of Imam Reza Hospital of Mashhad during 15 years (April 1995 to April 2010). All patients' data were collected from the questionnaires and then analyzed statistically. From 1814 suspected patients to be suffering from ectoparasites, 375 patients had scabies and, 99 suffer from pediculosis. The mean age of patients was 26/18 ± 17/68. The most common age of scabies was 10–19 (27/7%) and pediculosis 0–9(9/6%) (*P* value = 0.00). The highest incidence of pediculosis was in women (3.6%) and scabies in men (13.7%) (*P* value = 0.00). Pediculosis is more common in children (9/9%) and scabies in workers (32%)(*P* value = 0.00). Scabies and pediculosis were more prevalent in patients from Razavi Khorasan Province with 18.7% and 5%, respectively (*P* value = 0.08).

## 1. Introduction

Ectoparasitic infestation can induce persecutor diseases. Some of the common diseases of this group are pediculosis and scabies. Pediculosis and scabies are caused by ectoparasites; patients usually present with itching. Body lice are vectors of* Rickettsia prowazekii, Borrelia recurrentis*, and* Bartonella quintana,* the etiological agents of epidemic typhus, relapsing fever, and trench fever, respectively. Proper hygienic condition can prevent these diseases. Although these illnesses are not the concern of health care systems, they can cause high morbidity. Their incidence varied around the world depending on type and place of living. Ectoparasitic infestation can be as sporadic, endemic or epidemic [1].

*Pediculus *is a blood sucking parasite that is specific to humans.* Pediculus humanus var capitis* involved human head,* Phthirus pubis* involve genital area, and* Pediculus humanus var corporis* infest human body and use it as a warm place to live and feed*. Pediculus capitis* is the most common type in this group of ectoparasites especially in age groups of 3–11 years. Since 1970, the incidence of* Pediculus capitis* is increasing in the world [2].

*Sarcoptes* is an obligatory skin parasite and is important in dermatology.* Sarcoptes *usually involved hand skin including area between fingers and wrist, as well as elbow, feet, testis, and other sites of body [2].

The prevalence of* Pediculus capitis* in school children was reported by some studies as follows: Yasuj 11% [3], Babol 2.2% [4], Kerman 3.8% [5], Hamedan 1.3% [6], Turkey 6.8% [7], Korea 4.1% [8], and Egypt 12% [9]. In a study on Iranian prisoners, 9% of patients with dermatology complain hadpediculosis and 57% had scabies [10].

In poor communities the prevalence of scabies is reported more than 20% [11]. The prevalence of scabies was reported 0.4% in Turkey [7], 2.09% in Sari, and 1.3% [12] in Somehsara.

Since mentioned diseases are considered among important parasitic skin diseases and show the level of public health, by considering their high prevalence in our country and the necessity of identification of region of common infections, the dominant species in the region, and the mode of their transmission to human, we decided to report a 15-year period retrospective statistics study of patients who referred to parasitology laboratory of Imam Reza Hospital of Mashhad, Iran, one of the most important laboratories of east Iran.

## 2. Objects and Method 

In this cross-sectional study which was performed in February 2012, records of 1851 patients who had been suspicious of suffering from ectoparasitosis and had been referred to parasitology laboratory of Imam Reza Hospital of Mashhad during a 15-year period (from April 1995 to April 2010) were evaluated. In this study, diagnostic method forpediculosis was inspection and microscopic examination and for diagnosis of scabies direct examination from eruption and then microscopic examination had been used.

The data were collected from their health records by a researcher made questionnaire. Patient demographic data including age, gender, occupation, place of birth and place of living, and final diagnosis were collected. Data were analyzed by SPSS v 15, using Chi square test. *P* value less than 0.5 was considered significant.

## 3. Results 

In the present study, 1814 patients were assessed, 375 patients had scabies and 99 had pediculosis. The patients' minimum age was one month and maximum age was 97 years.* Sarcoptes* was more common among males with incidence of 13.7%, and pediculosis was more common among females with incidence of 7% (*P* = 0.00) (Table 1). The mean age of patients was 26.18 ± 17.68 years. The most common incidence of scabies and pediculosis was observed in age groups of 10–19 years and 0–9 years, respectively (*P* = 0.00) (Table 2).

Considering occupation, scabies was more common among workers with incidence of 32% and pediculosis was more prevalent among children with incidence of 9.9% (*P* = 0.00) (Table 3).

Regarding the place of living, the highest percentage of patients with* Sarcoptes* infestation were from Razavi Khorasan Province (18.7%), and the highest percentage of patients with pediculosis were from Razavi Khorasan Province as well (5%) (*P* = 0.08) (Table 1).

The laboratory results confirmed initial diagnosis, 23.7% of scabies and 45.1% of pediculosis (*P* = 0.00).

## 4. Discussion

Pediculosis is one of the common ectoparasitic infestations that are still considered as one of the health problems in the world [6, 7].

Our study revealed that ectoparasitic infestation is gender-dependent; the pediculosis is more common among females and scabies is more common among males. Many other studies have also shown that pediculosis is more common among females, which is similar to our results [8, 12]. It can be related to the women's hair length.

However, some of the researches about scabies have declared higher incidence in women rather than men [13]. Furthermore, the incidence of scabies in age groups of 31–40 and 41–50 is higher in women and in age groups 11–20 is higher in men [14]. These results are partly different from our findings. Poudat and Nasirian, in their study, reported similar prevalence among two the genders [10]. It seems that more studies about scabies prevalence in Iran have to be performed.

In the present study, the highest incidence of scabies was among age groups of 10–19 years, and the highest incidence of pediculosis was among age groups of 0–9 years. In Lassa et al.'s study, England has the highest incidence of scabies which is also reported among groups of 10–19 years [15] which is similar to our results. The maximal incidence of disease was reported under 10 years old by Amro in Palestine [16] but in rural area of Brazil greater prevalence was under 4 years old [17]. These differences could be related to differences in lifestyle and hygienic conditions in different societies. Another paper from Occupied Palestine showed the highest rate of pediculosis in age groups of 4–11 years [18] which is in concordance with our results.

In the present study, the maximal rate of scabies observed among patients who were workers can be related to lower income, poor hygiene, and low education and the highest rate of pediculosis observed among children can be due to poor hygiene in childhood. Sim reported that increased economic status and increased parental concern about children might have resulted in decrease of head lice infestation in Korea [13]. A study in Iran reported that children with unemployed fathers have more incidence of head pediculosis [12]. Another Iranian study on soldiers showed greater incidence of scabies among soldiers whose parents were farmers [19]. A Study in Bushehr showed that children, whose fathers are workers or unemployed, are more likely to have scabies [20]. As it was described our results are in agreement with these findings.

The prevalence of ectoparasites varied around the world depending on hygienic condition of the communities [4–7, 9–11, 21–23]. The prevalence of head pediculosis among primary school children was reported to be 11% in Yasuj [3], 2.2% in Babol [4], 3.8% in Kerman [5], and 1.3 % Bahar Hamedan [6].

The prevalence of scabies is reported to be more than 20% in communities with low socioeconomic status. Studies that had been performed in Iran reported the incidence of scabies among primary school children, 2.09% in Sari [22], and 1.3% in Someasara [21]. According to the present study, the greater incidence of scabies and pediculosis was observed among patients from Razavi Khorasan Province, 18.7 and 5% separately.

A study in Germany showed that the relation between initial diagnosis and final diagnosis varied depending on the used diagnostic method; in diagnosing scalp pediculosis, this relation is 90.5% when wet combing method was used, and it is 28.6% when the diagnostic method is visual inspection [24]. Laboratory findings confirmed initial diagnosis of scabies and pediculosis cases 23.7% of 45.1% respectively. In this study, diagnostic method forpediculosis was inspection and microscopic examination and, for diagnosis of scabies, direct examination from eruption and then microscopic examination had been used.

## 5. Conclusion

Scabies is a prevalent dermatologic disease in Iran and is transmitted from person to person or from dressing or bed sheets to others. In this study, the highest incidence of scabies among different occupations was observed in workers with 32% incidence and between genders it was more common among males with incidence of 28.1%. Therefore, it seems that education about the signs and transmission method of this disease to high risk groups will help greatly to reduce the prevalence of scabies and prevent probable future epidemy. Increasing knowledge of high risk people and having good hygiene are the proper methods for controlling scabies in the community.

We found the most common incidence of pediculosis in age groups of 0–9 years; regarding gender, it was more common between females with incidence of 7.3%. Considering scalp pediculosis transmission way which is usually head to head, it will justify its higher incidence in primary school children and female gender. The risk factors for pediculosis are long hair, crowded family, age, personal hygiene, and contact with infected person as well as education levels. Therefore, to prevent pediculosis, physical contact with infected people must be restricted, and sharing dress and bed must be prevented. Training people about* Pediculus* life cycle, correct treatment method, and the importance of washing cloths and bedding with warm water or dry cleaning for eradicating insect and its eggs is very valuable. Besides periodic assessment of school children regarding pediculosis is necessary. The authors believe that considering pediculosis high incidence in kindergarten and school children, educating parents and teachers is an important method for preventing and controlling pediculosis.

## Figures and Tables

**Table 1:** Frequency distribution of final diagnosis based on gender and place of living.

Final diagnosis	Gender		Place of living		
	Female	Male	Razavi Khorasan	North Khorasan	South Khorasan
Scabies					
Number	127	248	341	10	2
Percent	14.2	28.1	21.1	28.6	6.7
Pediculosis					
Number	65	34	92	2	3
Percent	7.3	3.8	5.7	5.7	10
Negative results*					
Number	702	602	1185	23	25
Percent	75.5	68.1	73.2	65.7	83.3

*Cases with final diagnosis except ectoparasites.

**Table 2:** Frequency distribution of final diagnosis based on age.

Age group			Final diagnosis					
	Scabies		Pediculosis		Negative		Total number	
	Number	Percent	Number	Percent	Number	Percent		
0–9	65	20.1	31	9.6	227	70.3	323	100
10–19	101	27.7	28	7.7	235	64.6	364	100
20–29	84	20.0	16	3.8	320	76.2	420	100
30–39	49	17.6	10	3.6	220	78.9	279	100
40–97	69	18.9	12	3.3	285	77.9	366	100

Total	368	21.0	97	55	1287	73.5	1752	100

**Table 3:** Frequency distribution of final diagnosis based on occupation.

Occupation			Final diagnosis					
	Scabies		Pediculosis		Negative		Total number	
	Number	Percent	Number	Percent	Number	Percent		
Employee	24	16.2	6	4.1	118	79.7	148	100
Worker	16	32.0	2	4.0	32	64.0	50	100
Self employed	73	30.8	7	3.0	157	66.2	237	100
Housewife	58	11.6	22	4.4	419	84.0	499	100
Student	98	27.8	23	6.5	231	65.6	352	
Child	52	20.6	25	9.9	176	69.9	253	100
Retired/unemployed	44	22.4	11	5.6	141	71.9	196	100

Total	365	21.0	96	5.5	1274	73.4	1735	100
